# Supplementary material for: In situ atomistic insight into the growth mechanisms of single layer 2D transition metal carbides
Source: Nat Commun. 2018 Jun 11;9:2266. doi: 10.1038/s41467-018-04610-0 (PMC5995835; doi:10.1038/s41467-018-04610-0)
Supplement: Supplementary file 1 — Supplementary Information [file 41467_2018_4610_MOESM1_ESM.pdf]

Supplementary Information for

In situ atomistic insight into the growth mechanisms of single layer 2D transition metal carbides

Sang *et al.*

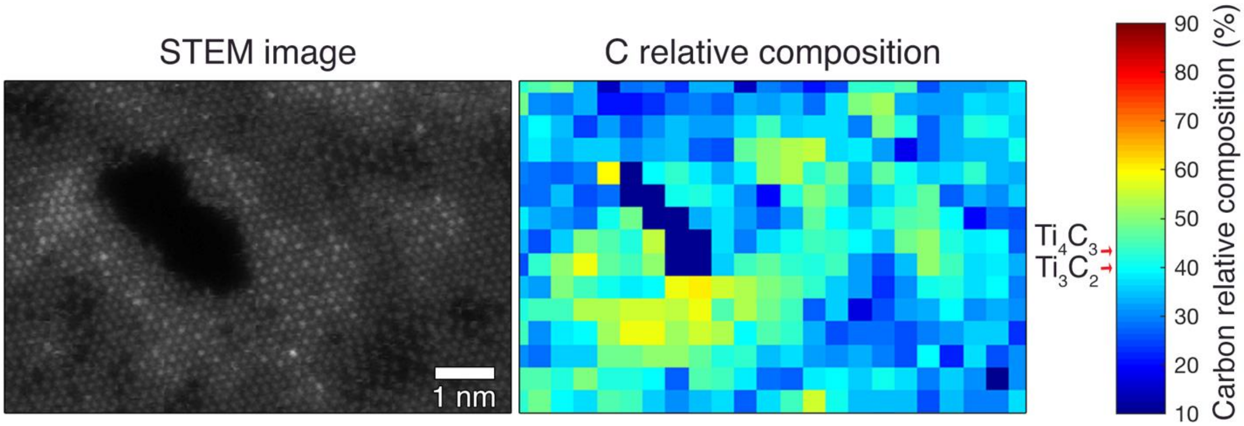

Supplementary Figure 1 | Electron energy loss spectroscopy elemental mapping from the adlayer areas. Atomic resolution STEM image (left) acquired from a single layer Ti_3C_2 flake after homoepitaxial growth, and the corresponding C relative composition map obtained from EEL spectrum imaging. Blue areas have low C composition, while yellow and red areas have high C composition. The adlayer areas generally show higher C concentration.

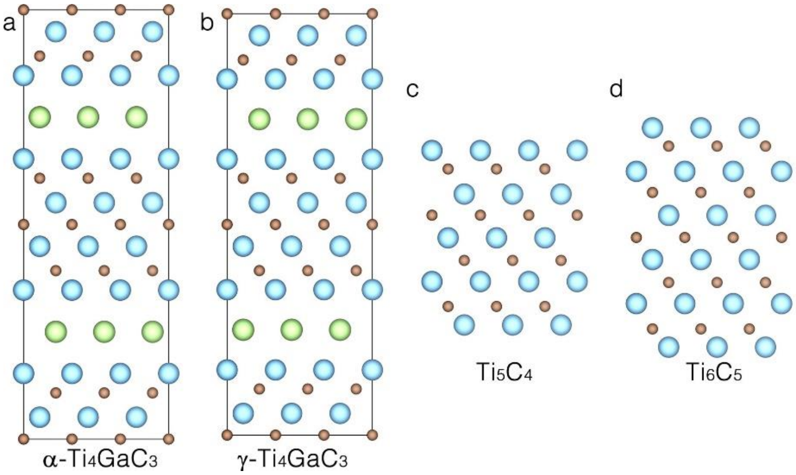

Supplementary Figure 2 | Stacking of *h*-TiC adlayer on Ti_3C_2. a-b, Side view of crystal structure of α-Ti_4GaC_3 (**a**) and γ-Ti_4GaC_3 (**b**). The structure of Ti_4C_3 block is the same as we predicted for *h*-TiC adlayer on Ti_3C_2. **c-d,** Side view of structure of Ti_5C_4 (**c**) and Ti_6C_5 (**d**). The repeating *CAB* stacking sequence is clear. Light green, Ga; Blue, Ti; gray, C.

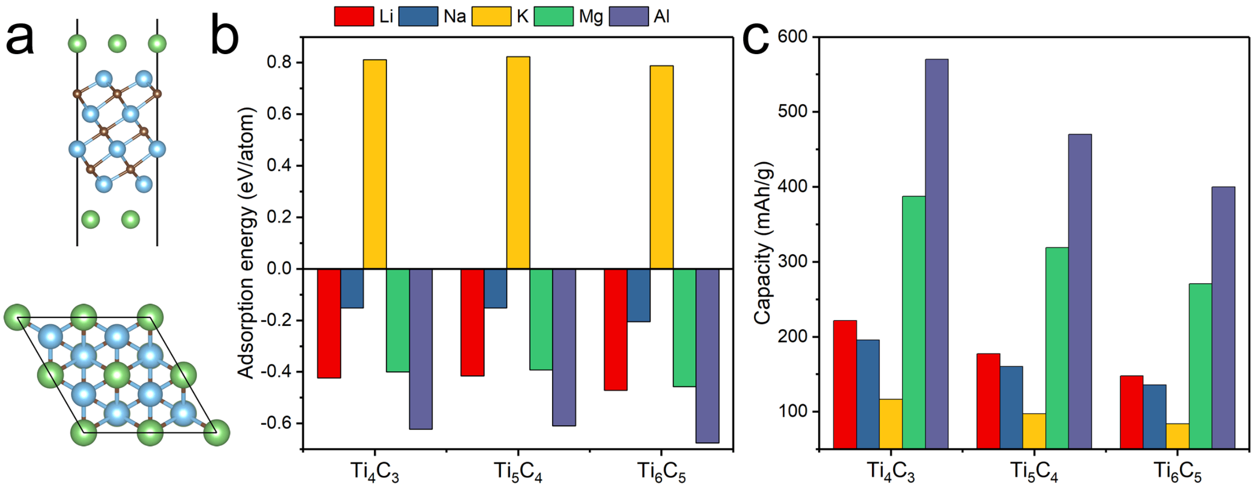

Supplementary Figure 3 | Theoretical adsorption energies and capacities for Ti_4C_3 and Ti_5C_4. **a**, Side and top views of ion adsorption on Ti_4C_3 nanosheets. **b**, Theoretical adsorption energies of Ti_4C_3, Ti_5C_4, and Ti_6C_5 for different metal ions: Li, Na, K, Mg, and Al. K^+ can only form a partial 2/3 layer as observed in Ti_2C and Ti_3C_2. **c**, Theoretical capacities of Li, Na, K, Mg, and Al ions on Ti_4C_3, Ti_5C_4, and Ti_6C_5.

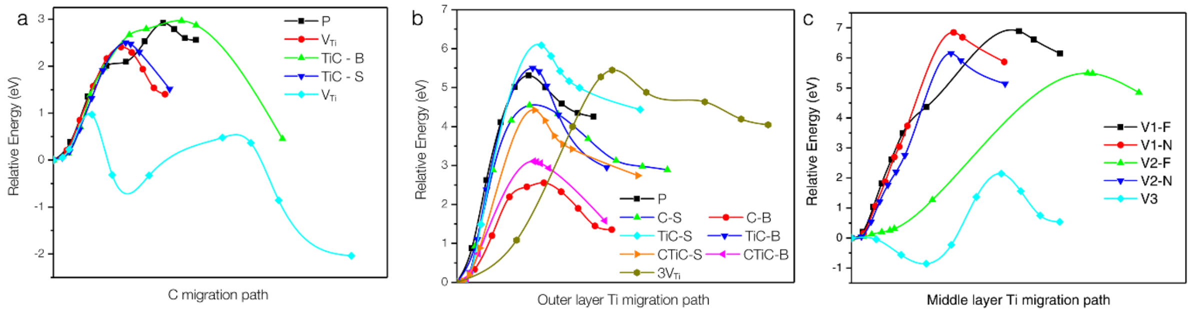

Supplementary Figure 4 | Migration energy path of Ti and C atoms from the bulk to the Ti_3C_2 surface. a-c, Calculated migration energy path from body to surface for C atom (**a**), outer layer Ti atom (**b**), and middle layer Ti atom (**c**).

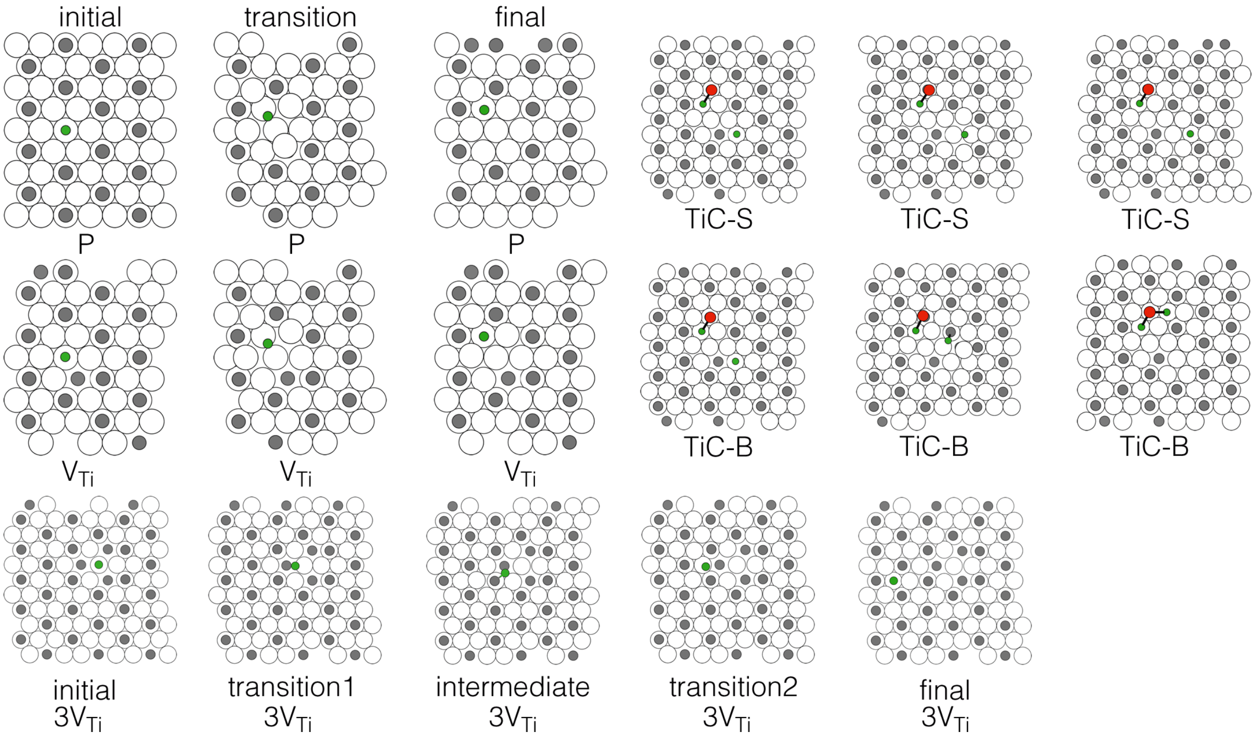

Supplementary Figure 5 | C migration from body to Ti_3C_2 surface through different pathways. Initial, transition, and final states are shown for different migration pathways. Here "P" denotes pristine Ti_3C_2, while "TiC" means one Ti and one C atoms have already diffused to Ti_3C_2 surface and formed TiC dimer. "-S" indicates that after migrating to the Ti_3C_2 surface the C atom bonds with the TiC dimer, and "-B" means after migrating to the Ti_3C_2 surface the C atom is not bonded with the TiC dimer. "V_{Ti}" denotes Ti vacancy. White, Ti of Ti_3C_2; gray, C of Ti_3C_2; red, moving Ti; green, moving C. See Supplementary Fig. 4a for the energy plot.

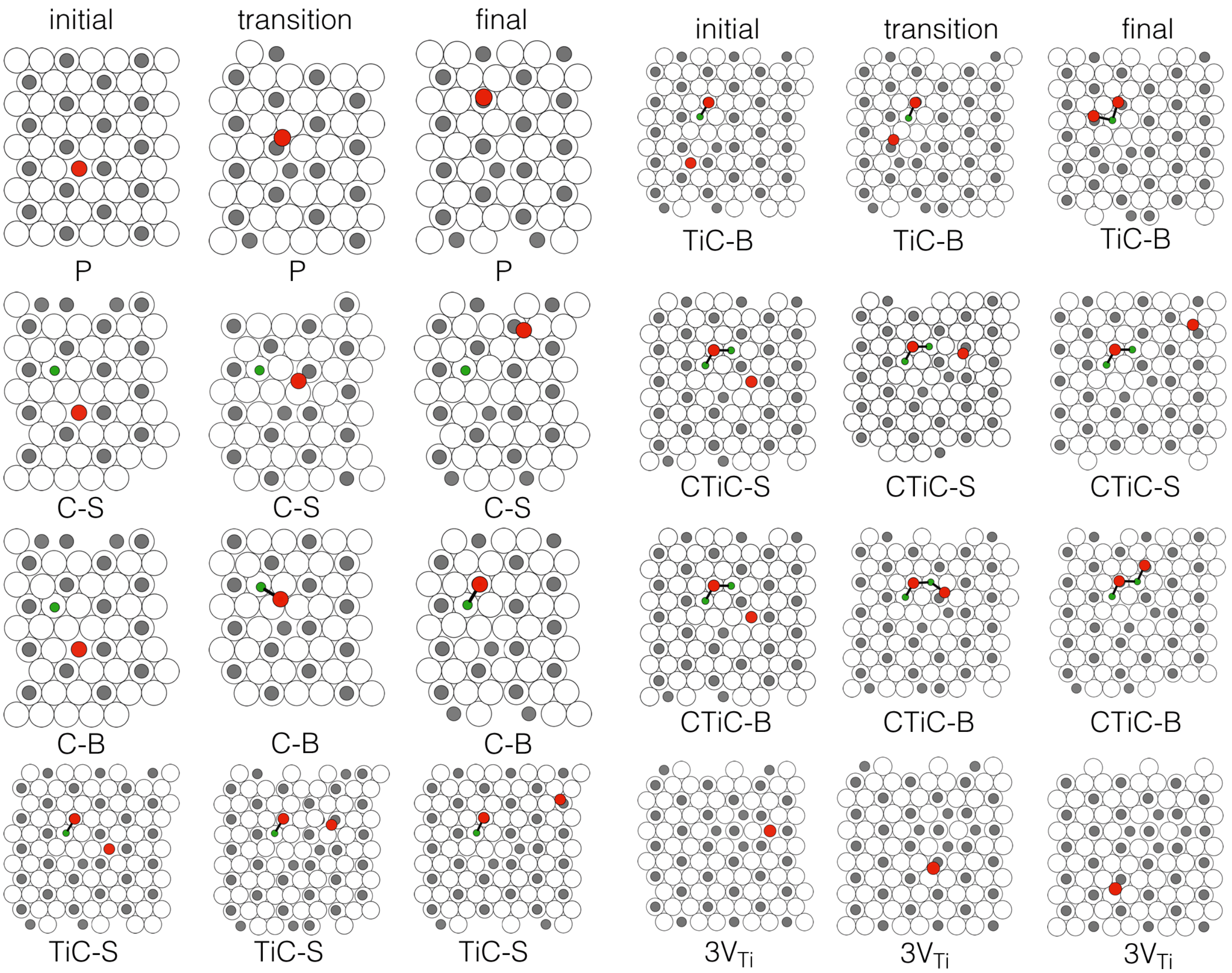

Supplementary Figure 6 | Different migration pathways for outer layer Ti atoms from body to Ti_3C_2 surface. Initial, transition, and final states are shown for different migration pathways. Here "P" denotes pristine Ti_3C_2 surface, while "C", "TiC", and "CTiC" means originally the Ti_3C_2 surface has one C adatom, a TiC dimer formed by one C adatom and one Ti adatom, and a CTiC trimer formed by two C adatoms and one Ti adatom, respectively. "-S" indicates that the Ti atom bonds with surface adatoms after migrating to the Ti_3C_2 surface, and "-B" means the Ti atom is not bonded with surface adatoms after migrating to the Ti_3C_2 surface. "$3V_{Ti}$" means a vacancy cluster of three Ti vacancies on the surface. White, Ti of Ti_3C_2; gray, C of Ti_3C_2; red, moving Ti; green, moving C. See Supplementary Fig. 4b for the energy plot.

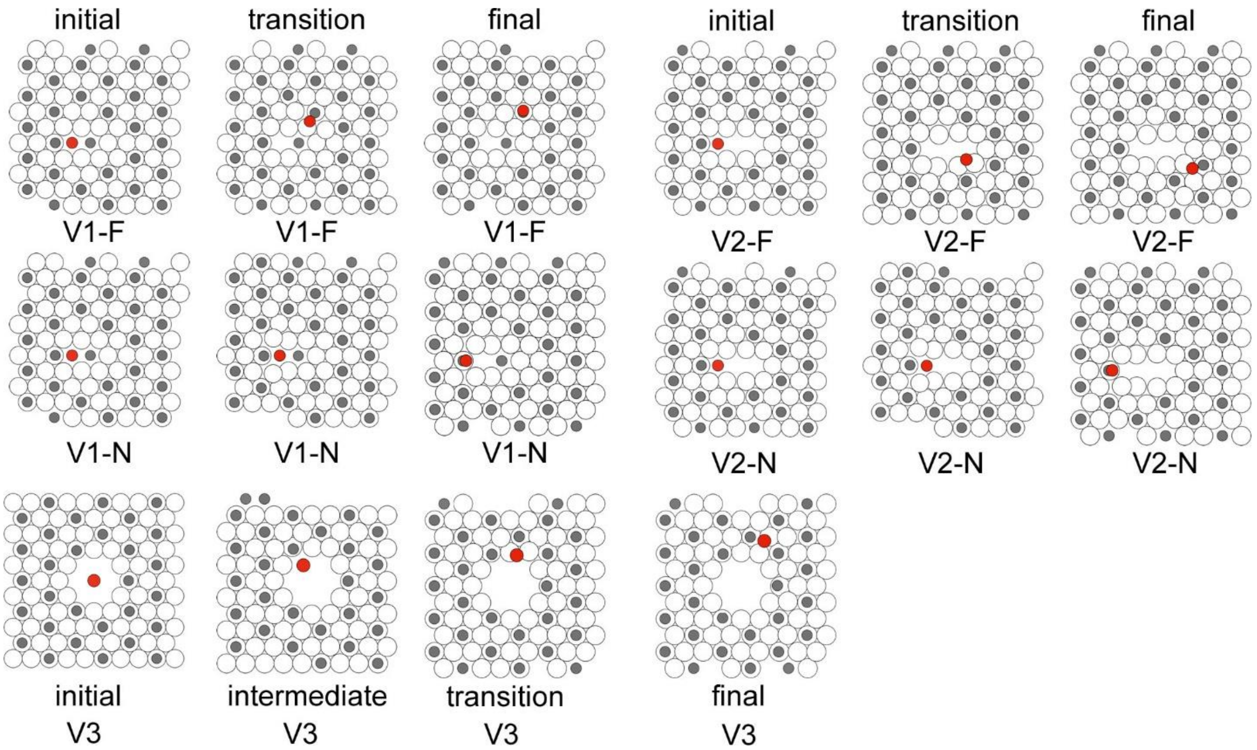

Supplementary Figure 7 | Different migration pathways for middle layer Ti atoms from body to Ti_3C_2 surface. Initial, transition, and final states are shown for different pathways. Here "V" denotes the pinhole defect of Ti_3C_2. "1", "2", and "3" indicate the number of Ti vacancy in the outmost Ti layer, and therefore the size of the pinhole. "N" and "F" mean Ti atom diffuses to the first nearest and second nearest adsorption site on Ti_3C_2 surface, respectively. White, Ti of Ti_3C_2; gray, C of Ti_3C_2; red, moving Ti; green, moving C. See Fig. S4c for the energy plot.

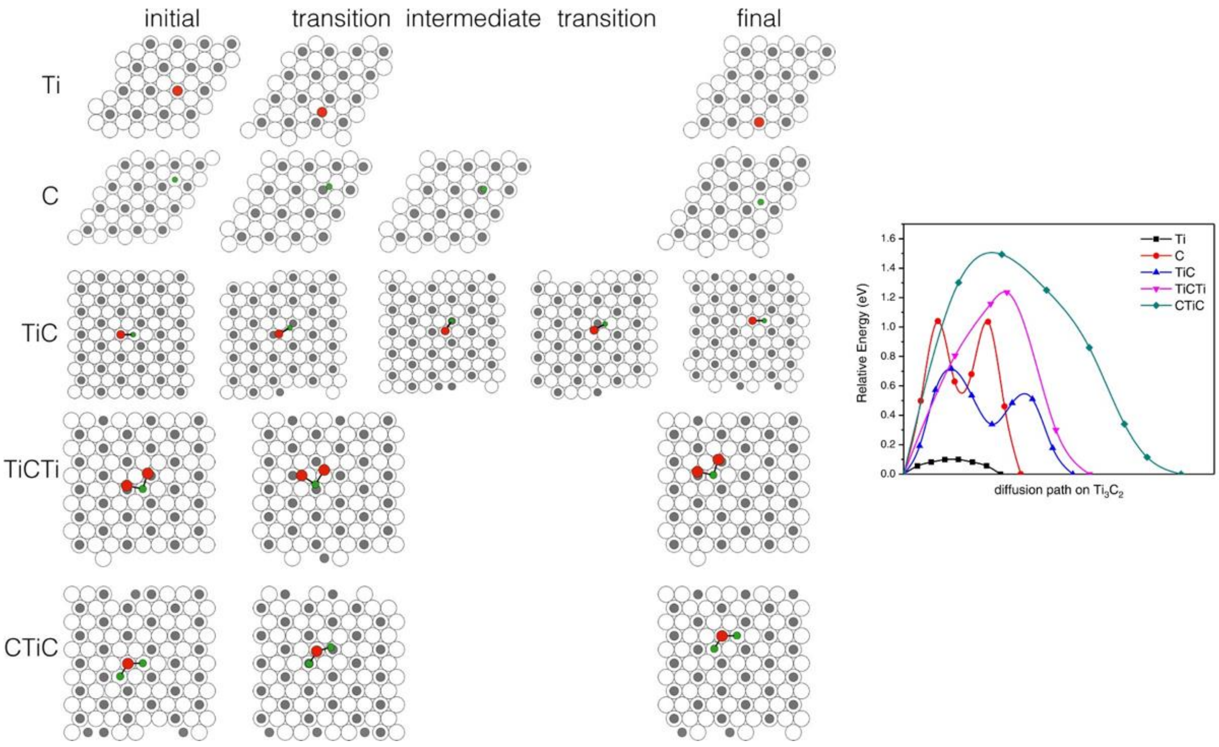

Supplementary Figure 8 | Diffusion paths of Ti atom, C atom, TiC dimer, and trimers on Ti_3C_2 surface. Initial, transition, and final states for a Ti atom, a C atom, a TiC dimer, a TiCTi trimer, and a CTiC trimer to diffuse on the Ti_3C_2 surface. White, Ti of Ti_3C_2; gray, C of Ti_3C_2; red, moving Ti; green, moving C. The energy barrier of the diffusion paths is shown on the right.

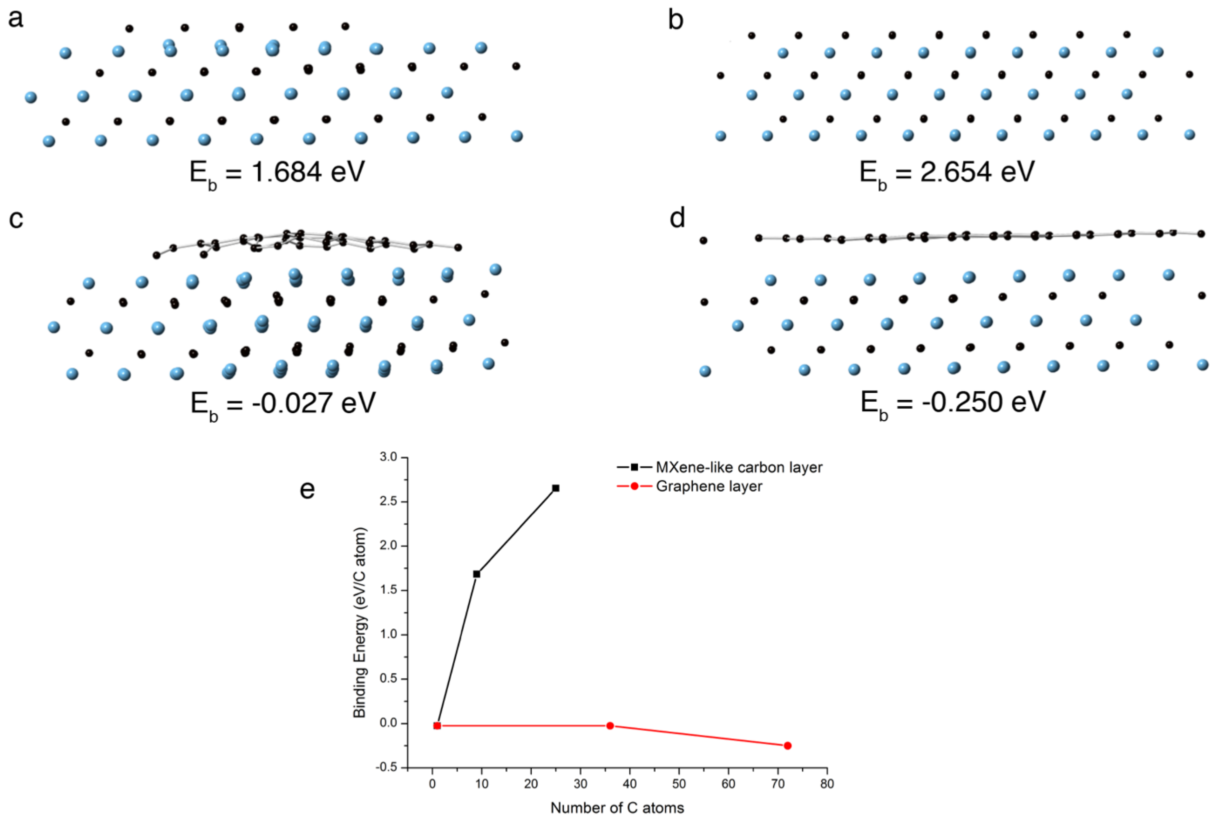

Supplementary Figure 9 | Formation energy of graphene-like C layer versus MXene-like C layer. a-d, Using density functional theory (DFT), optimized crystal structure along the *a* axis of an additional MXene-like C layer of (**a**) 10 C atoms and (**b**) 25 C atoms, on Ti_3C_2 substrate. Optimized crystal structure of an additional graphene-like C layer of (**c**) 36 atoms and (**d**) 72 atoms. **e,** Binding energy per C atom for MXene-like C layer and graphene layer as a function of number of C atoms. The binding energy of MXene-like C layer on Ti_3C_2 surface is 2.65 eV/atom, which is much higher than that of graphene ~ -0.25 eV/atom. This suggests that sequential layer-by-layer growth is unlikely because If C atoms form a layer first, they should prefer the thermodynamically more stable graphene-like morphology. Although Ti atoms can grow on graphene, the theoretical Ti-Ti bond length is 2.46 Å [1], which is shorter than that of Ti_4C_3 ~ 3.09 Å. Such lattice mismatch pattern has not been observed experimentally, which therefore excludes the possibility of a growth mode that a carbon layer grows first and the titanium layer grows on top of the carbon layer.

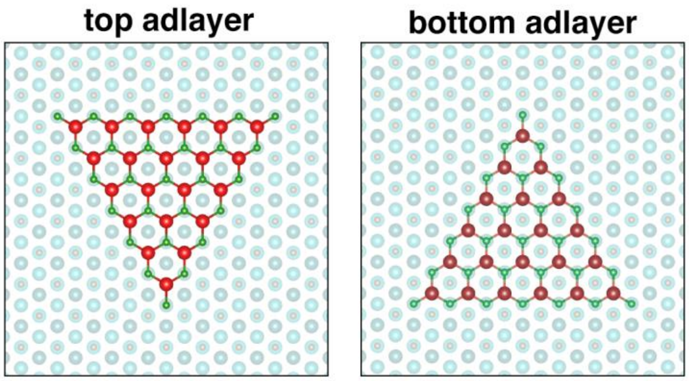

Supplementary Figure 10 | Different orientations of triangular adlayers on top and bottom surfaces. Orientation of triangular adlayers on the top surface (top adlayer) and bottom surface (bottom adlayer) assuming both adlayers are terminated with carbon-oriented zigzag edges.

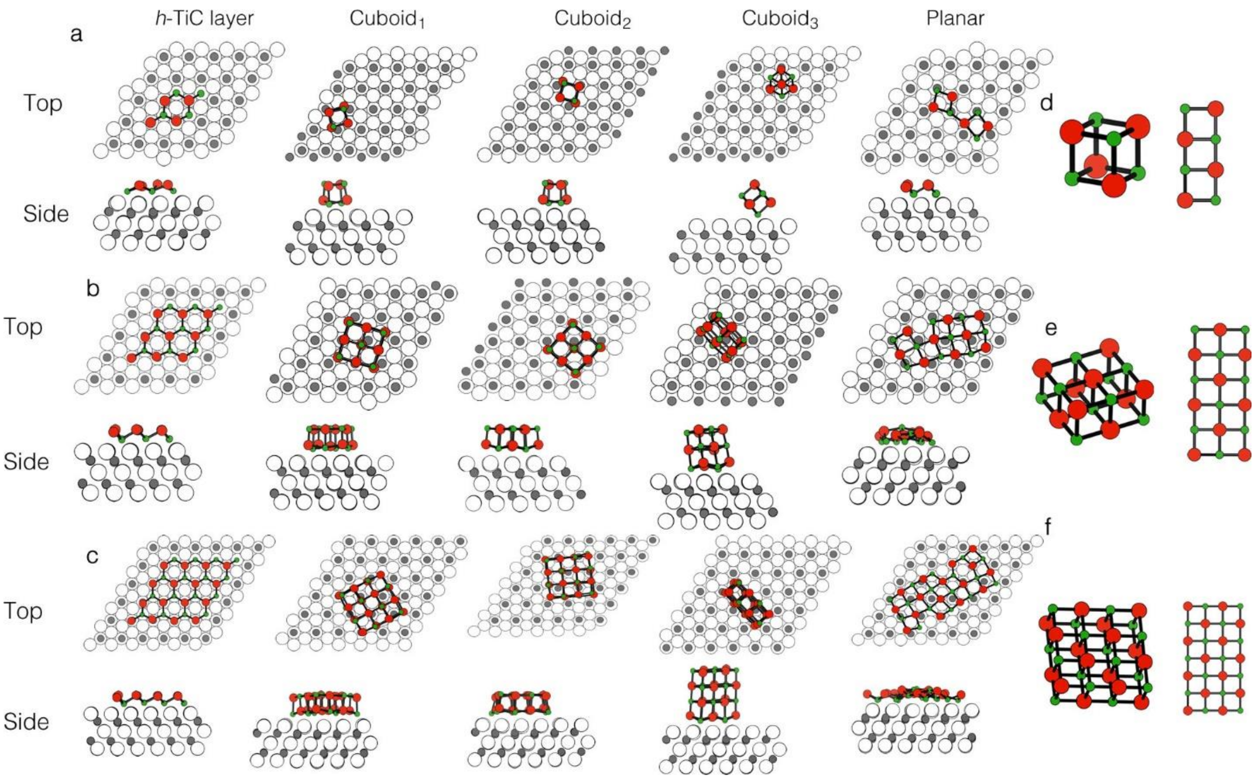

Supplementary Figure 11 | Crystal structure models of TiC nanoparticles (NP) on bare Ti_3C_2. a-c, Optimized structure of *h*-TiC adlayer, cuboid and planar TiC NPs on Ti_3C_2 with different size. **d-f,** The crystal structures of isolated TiC NPs.

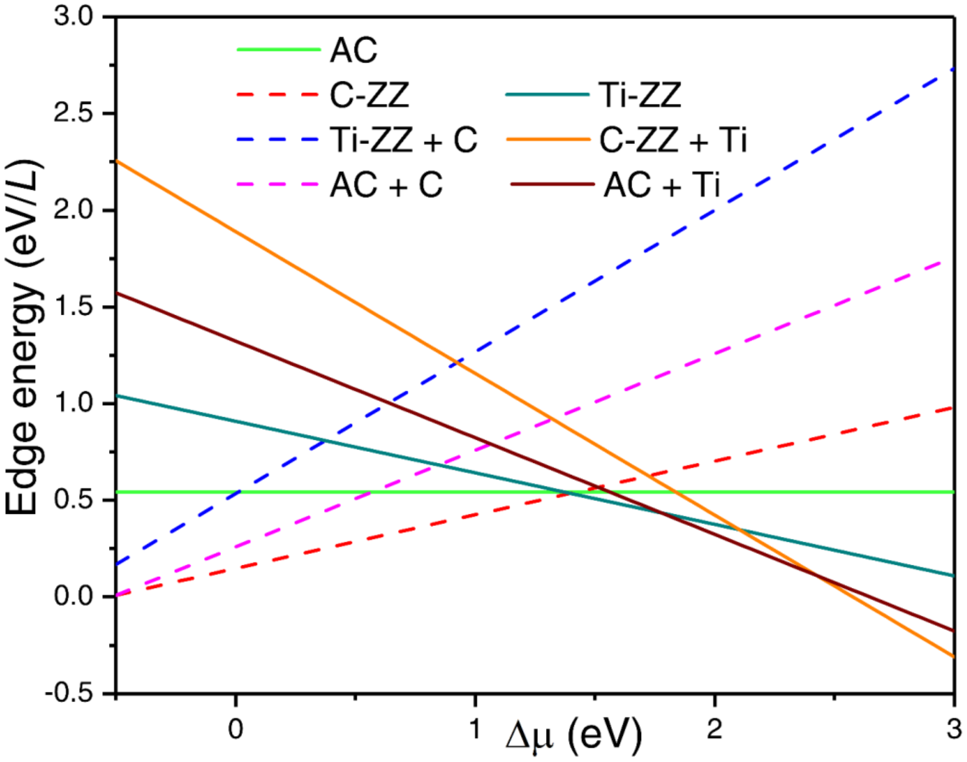

Supplementary Figure 12 | Edge structure and equilibrium shape of *h*-TiC layer. Edge energies of *h*-TiC on Ti_3C_2 as a function of chemical potential difference between Ti and C, $\Delta\mu$. *L* is the length of the Ti_4C_3 unit cell. In the main text, we discuss the edge structure stability based on the Ti chemical potential difference $\Delta\mu_{Ti}$, where we assume the Ti source is from Ti bulk or Ti_3C_2. Since we propose that 2D *h*-TiC should also form by feeding Ti and C atoms in gas sources, we extend our study on the stability of edge structures to a wider chemical potential range as a function of the chemical potential difference between Ti and C, $\Delta\mu = (\mu_{Ti} - \mu_C)/2$, which satisfies $\mu_{Ti} + \mu_C = \mu_{TiC}$. Therefore, the Ti-rich and C-rich condition in the main text is $\Delta\mu = 1.502$ and $\Delta\mu = -0.041$, respectively. By increasing the chemical potential of Ti, the Ti-terminated edge becomes more stable. The Ti-terminated AC is stable between 1.779 and 2.432 eV. After that, Ti-terminated ZZ_C dominates. On the other hand, by decreasing the chemical potential of Ti, the C-terminated edge is more stable. We notice that the formation energy of the edge structure becomes negative when $\Delta\mu$ is below -0.51 eV or above 2.56 eV, suggesting the edge structure is not stable and it will undergo a surface reconstruction. Therefore, to grow 2D *h*-TiC using bottom-up methods, it is better to maintain the chemical potential in slightly Ti-rich condition.

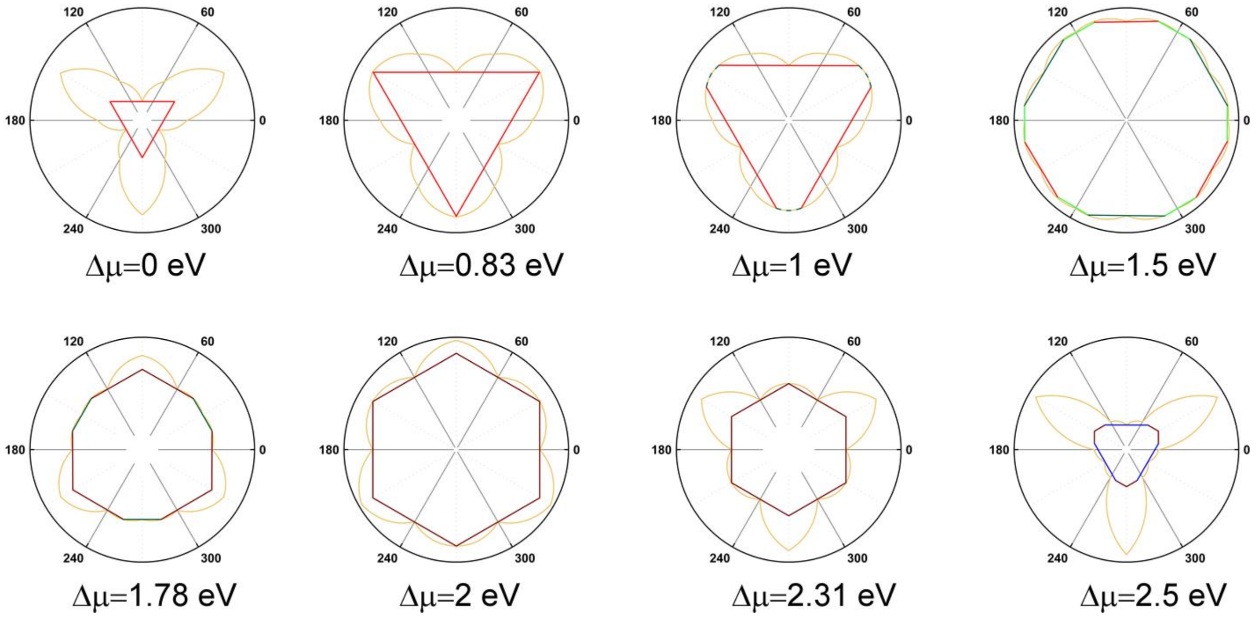

Supplementary Figure 13 | The equilibrium shape of *h*-TiC on Ti_3C_2 at varying Δμ. The colored outlines represent the type of edge structure. When the Ti-terminated ZZ_Ti edge dominates, *h*-TiC adlayer may show a hexagonal morphology.

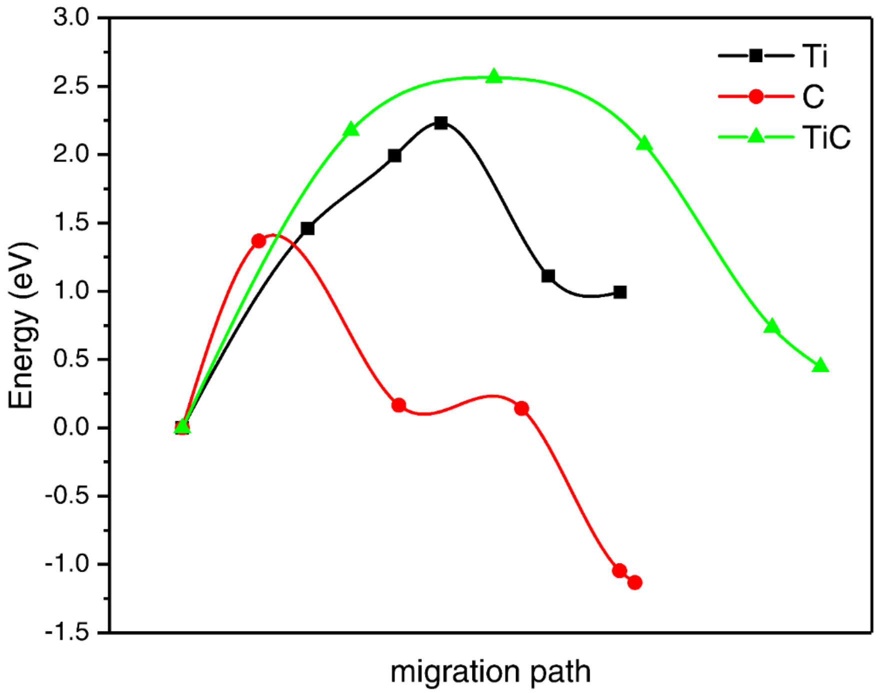

Supplementary Figure 14 | Step-edge energy barriers. Migration energy paths of a Ti atom, a C atom, and a TiC dimer climbing up to the top of a *h*-TiC adlayer. The energies barriers here are lower than energy barriers for Ti and C atom to move from the body to the surface, while the energy barriers are higher than the diffusion barriers of Ti and C adatoms on the Ti_3C_2 surface.

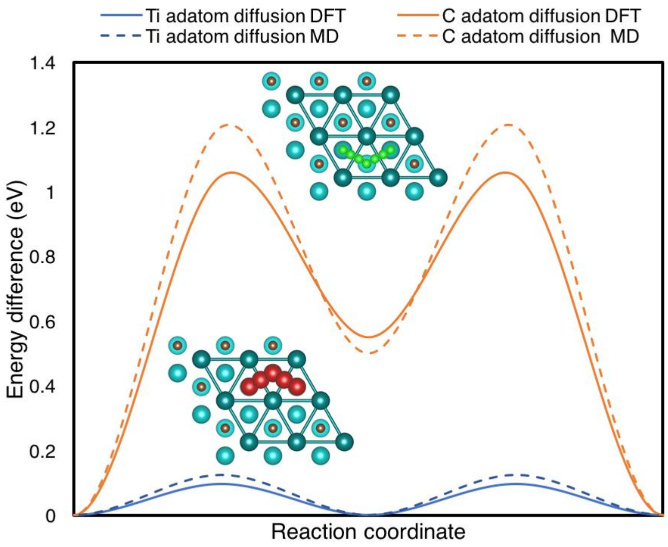

Supplementary Figure 15 | Comparison between diffusion barriers predicted from ReaxFF and DFT calculation. Diffusion barrier of Ti and C adatoms on Ti_3C_2 surface using DFT (solid line) and ReaxFF (dashed line). The red atoms are Ti adatoms while the green atoms are C adatoms. The agreement between DFT and ReaxFF is excellent. Therefore, simulations requiring large number of atoms were performed using ReaxFF instead of DFT.

Supplementary Table 1. Calculated formation energies of *h*-TiC adlayer, cuboid nanoparticles (NP), and planar TiC NPs on Ti_3C_2 surface reference to the TiC bulk (see Supplementary Fig. 9 for the crystal structures). The *h*-TiC adlayer always has the lowest formation energy. For cuboid TiC NPs, the formation energy decreases as the NP size increases due to the increased interacting area with Ti_3C_2 surface.

N_{TiC}	*h*-TiC layer (eV/TiC)	$Cuboid_1$ (eV/TiC)	$Cuboid_2$ (eV/TiC)	$Cuboid_3$ (eV/TiC)	Planar NP (eV/TiC)
4	0.86	1.91	1.99	2.04	1.30
9	0.57	1.60	1.60	1.66	1.02
16	0.44	1.11	1.16	1.54	0.96

Supplementary References

1 Zanella, I., Fagan, S. B., Mota, R. & Fazzio, A. Electronic and Magnetic Properties of Ti and Fe on Graphene. *The Journal of Physical Chemistry C* **112**, 9163-9167 (2008).
