# Supplementary material for: In situ atomistic insight into the growth mechanisms of single layer 2D transition metal carbides
Source: Nat Commun. 2018 Jun 11;9:2266. doi: 10.1038/s41467-018-04610-0 (PMC5995835; doi:10.1038/s41467-018-04610-0)
Supplement: Supplementary file 3 — Description of Additional Supplementary Files [file 41467_2018_4610_MOESM3_ESM.docx]

**Description of Additional Supplementary Files**

File Name: Supplementary Movie 1

Description: A single-layer Ti_3_C_2_T_x_ flake is stable under the electron beam at room temperature.

File Name: Supplementary Movie 2

Description: Structural evolution of single-layer Ti_3_C_2_T_x_ at 500 °C.
